# Distinct NF-kB Regulation Favors a Synergic Action of Pevonedistat and Laduviglusib in B-Chronic Lymphocytic Leukemia Cells Ex Vivo

**DOI:** 10.3390/cancers17030533

**Published:** 2025-02-05

**Authors:** Víctor Arenas, Jose Luis Castaño, Juan José Domínguez, Lucrecia Yáñez, Carlos Pipaón

**Affiliations:** 1 Molecular Hematology Laboratory, Marqués de Valdecilla Research Institute, 39008 Santander, Spain; victor.arenas@idival.org (V.A.); jose.castano@idival.org (J.L.C.); 2 Hematology Department, Marqués de Valdecilla University Hospital, 39008 Santander, Spain; juanjose.dominguez@scsalud.es (J.J.D.); lucrecia.yanez@scsalud.es (L.Y.)

**Keywords:** chronic lymphocytic leukemia, MLN4924, CHIR-99021, NEDD8, GSK-3ß, NF-kB

## Abstract

**Background/Objectives:** Chronic lymphocytic leukemia (CLL) remains an incurable B-cell malignancy. B-CLL cells exhibit an extended lifespan in part due to the activation of survival pathways such as NF-kB. A crosstalk between NF-kB and GSK-3β pathways has been reported. NF-kB has also been identified as a primary target of the NEDD8-activating enzyme inhibitor MLN4924. Our objective was to investigate potential synergies of MLN4924 with other NF-kB-targeting agents for the treatment of CLL and elucidate the mechanisms of action underlying this pathway regulation. **Methods:** To assess the cytotoxic efficacy of the combined ex vivo treatment with CHIR-99021 and MLN4924, we employed 7-AAD staining and XTT viability assays on primary samples from CLL patients. Subsequently, we conducted various analyses to identify the molecular mechanisms underlying the cytotoxic effects of this combination. **Results:** We discovered a discrepancy between the mRNA and protein levels of IkBɑ and provided evidence of translational control over its expression. This observation may explain why, unlike other cell types, B-CLL cells did not activate NF-kB signaling following inhibition of GSK-3ß. Furthermore, we describe a synergistic effect between a specific GSK-3ß inhibitor, CHIR-99021/Laduviglusib, and the NEDD8-activating enzyme inhibitor MLN4924/Pevonedistat, at doses that only slightly affect healthy B cell viability ex vivo. We investigated the molecular basis of this co-induction of cell death by analyzing the alterations in apoptosis-related gene expression. We found that the combinational treatment enhances a reduction in BCL2 mRNA expression levels, providing an alternative approach for BCL-2 inhibition in CLL that could have therapeutic implications for the treatment of refractory CLL cases. **Conclusions:** our findings revealed a unique interaction between GSK-3ß and NF-kB pathways in CLL and their regulation of BCL2 expression.

## Simple Summary

Although a central role has been attributed to NF-kB signaling in chronic lymphocytic leukemia (CLL), numerous observations suggest a complex regulation of this pathway. In this study, we provide evidence of a previously unreported translational regulation of the key endogenous regulator of the pathway: IkBα. This process elucidates the discrepancy between IkBα mRNA and protein levels and explains why GSK-3β inhibition fails to activate NF-kB signaling and, consequently, cell survival in these cells. Based on these findings, we explore a treatment strategy for CLL cells ex vivo, combining GSK-3β and NEDDylation inhibition. We observed a potentiation of their individual actions and provide data suggesting a potential mechanism through the inhibition of BCL2 expression. These observations support further research for the development of novel therapies for CLL.

## 1. Introduction

Chronic lymphocytic leukemia (CLL) is the most common lymphoproliferative disorder in Western countries [1]. Despite ongoing research, its molecular basis remains largely unknown. CLL is characterized by an accumulation of mature B cells in the bloodstream, lymphoid organs, and bone marrow, which can eventually impair the production of other blood lineages. This accumulation results in part from a failure in the apoptotic mechanisms of CLL tumor cells due to intrinsic and intercellular signaling mechanisms. Consequently, current therapies target the B-cell receptor (BCR)-activated pathways or block the action of antiapoptotic proteins [2].

NF-kB, a transcription factor involved in inflammation and survival, is constitutively active in various tumors, including CLL, through distinct pathways [3]. The canonical pathway is regulated by the degradation of its inhibitor, IkB, which binds and sequesters NF-kB in the cytoplasm. Phosphorylation of IkB at serines 32 and 36 by a cascade initiated by extracellular stimuli triggers its ubiquitination and degradation at the proteasome, releasing NF-kB transcription factor to translocate into the nucleus and activate survival genes [4]. NF-kB is a dimer composed of various proteins. The dimer activated by the canonical pathway typically consists of p65 (RelA) and p50. NF-kB-dependent transcription is also directly modulated by non-NF-kB pathways [5]. For instance, GSK-3β has been reported to phosphorylate the highly transactivating p65/RelA subunit at serine 468 in unstimulated cells [6], allowing it to interact with the orphan receptor NURR1/NR4A2, which recruits transcriptional corepressors [7]. GSK-3ß has been shown to accumulate in the nucleus of B-CLL cells, and its inhibition has been associated with epigenetic changes in the promoters of the antiapoptotic genes BCL2 and XIAP, which preclude p65 from binding to them [8]. To prevent continuous signaling in healthy cells, the NF-kB pathway upregulates the transcription of its own inhibitor IkBα, among other feedback mechanisms [9]. IkBα is also tightly regulated by post-transcriptional mechanisms. Since IkBα is ubiquitinated by a Cullin-RING E3 ligase, several groups have explored its stabilization by modulating the NEDDylation of its Cullin module. Indeed, an inhibitor molecule (MLN4924-Pevonedistat) of the NEDD8-activating enzyme NAE has been utilized to block NF-kB in various tumor cells and is currently undergoing clinical trials [10]. MLN4924 has been demonstrated to reduce the viability of B-CLL cells and synergizes with inhibitors of BCR-activated pathways [11]. The results of a clinical trial investigating the combination of MLN4924/Pevonedistat with ibrutinib, a Bruton’s kinase inhibitor, in B-cell non-Hodgkin lymphoma patients have recently been published [12]. Our research group observed a general increase in ubiquitin-like post-translational modifications (including ubiquitination, NEDDylation, and ISGylation) and explored their impact on the function of p53 and BCL-2 in CLL [13]. Notably, IkBα was not detected as aberrantly modified in this screening [14].

In addition to the ubiquitin-proteasome regulation of its stability mentioned earlier, translational control of NFKBIA mRNA that encodes for IkBα has been reported as a rapid mechanism for cells to combat viral infections by activating the production of type-I interferon through NF-kB signaling [15]. Generally, translational control is exerted at the initiation step, where the eIF4F complex facilitates the interaction of the 5′ end of mRNAs with the ribosome. Given that many tumors exhibit dependency on mRNA translation for their transformation, several research groups are investigating mRNA translation inhibition as a potential cancer therapy [16,17]. In CLL, inhibition of eIF4A reduced BCR-induced global translation, as well as specifically that of *MYC* and *MCL1* [18]. Synthetic rocaglates have been identified as potent eIF4A inhibitors and their potential for treating MYC-driven lymphomas has been evaluated [19].

In this study, we investigated several mechanisms that regulate NF-kB signaling in CLL cells in an effort to identify novel strategies for cooperatively inhibiting its activity. We observed a discrepancy between the mRNA and protein expression of IKBɑ, which could be attributed to the elevated mRNA translation observed in these cells. Additionally, we provided evidence demonstrating that GSK-3ß inhibition did not enhance NF-kB signaling or survival in B-CLL cells, as it did in other cell lines. Conversely, NEDDylation inhibition potentiated the effect of the GSK-3ß inhibitor CHIR-99021/Laduviglusib in inducing B-CLL cell death. Furthermore, our findings revealed a disparity in the response of *BCL2* or *BIRC3* and that of NF-kB target genes to the combinational treatment with CHIR-99021 and MLN4924 in these cells.

## 2. Materials and Methods

### 2.1. Patient Samples

Peripheral blood samples obtained from patients with chronic lymphocytic leukemia (CLL) were utilized after obtaining informed consent. Ethical approval was granted by the Ethics Committee of Cantabria (Resolution 2017.261). Peripheral blood mononuclear cells (PBMCs) were isolated via centrifugation in a Ficoll (GE Healthcare, Uppsala, Sweden) gradient. As an inclusion criterion, CLL patients were selected with a higher-than- 85% lymphocytic infiltration (i.e., more than 85% of the leukocytes are lymphocytes). Patients currently undergoing treatment were excluded from the study. The primary characteristics of the patients are presented in Table 1. B cells from healthy donors were isolated from buffy coats after obtaining informed consent using the MACSprep HLA B Cell Isolation Kit (Miltenyi, Bergisch Gladbach, Germany).

### 2.2. Cell Lines and Transfection

HEK293T human embryonic kidney cells were maintained in DMEM medium (BioWest, Nuaillé, France) supplemented with 10% heat-inactivated fetal calf serum and cultured in a humidified 5% CO_2_-containing atmosphere at 37 °C. PBMCs were cultured overnight in RPMI (Avantor, Llinars del Vallès, Spain) supplemented with 10% heat-inactivated fetal bovine serum in the presence of the indicated inhibitors or an equivalent volume of diluent (DMSO). HEK293T cells were transfected using the calcium phosphate method with 3 µg of total DNA in 6-well plates.

### 2.3. Cell Death and Viability Assays

Peripheral blood mononuclear cells (PBMCs) derived from patients with chronic lymphocytic leukemia (CLL) were ex vivo treated with MLN4924, BAY11-7082, ABT-199/Venetoclax (Cayman Chemical, Ann Arbor, MI, USA) or 1 µM CHIR-99021 (MedChemExpress, Sollentuna, Sweden) as indicated. Cell death was assessed by flow cytometry after staining with 7-AAD (Enzo Biochem, Farmingdale, NY, USA) in a DxFLEX Flow Cytometer (Beckman Coulter, Indianapolis, IN, USA). For viability assays, an XTT cell viability kit (Biotium, Fremont, CA, USA) was utilized in 96-well plates. The synergistic effect of the combined drug treatments was evaluated using the SynergyFinder+ web page (https://synergyfinder.org, accessed on 9 December 2024).

### 2.4. Plasmids and Interference Constructs

The open reading frame of GSK-3ß was amplified from a human retrotranscribed RNA sample using the oligos: 5′-GGGG-AGATCT-ATGTCAGGGCGGCCCAGAACC-3′ and 5′-GGGG-CTCGAG-TGTTCAGGTGGAGTTGGAAGC-3′. The PCR product was ligated between the BamHI and XhoI sites of pCMVTag2B plasmid. S9A and K85A point mutants were generated by partial PCR amplifications of the wild type clone with oligos carrying the mutations and cloned in the same vector. Similarly, p65/RelA was cloned into the same plasmid after amplification from a human RT using the oligos: 5′-GGGG-GGATCC-ATGGACGAACTGTTCCCCCTC-3′ and 5′-GGGG-CTCGAG-TTAGGAGCTGATCTGACTCAG-3′. CRISPR/Cas9 constructs for SENP8 (pCMV Cas9-GFP SENP8 #20 and #22) (Merck, Darmstadt, Germany) were transfected into HEK-293T cells by the calcium phosphate method and GFP-positive cells were sorted by flow cytometry. The TopFlash reporter plasmid was constructed by annealing the oligos 5′-TCGAG-AAGATCAAAGGGGGTAAGATCAAAGGGGGTAAGATCAAAGGG-A-3′ and 5′-AGCTT-CCCTTTGATCTTACCCCCTTTGATCTTACCCCCTTTGATCTT-C-3′ and ligating them between the XhoI and HindIII sites of the pGL2-TATA plasmid. The pBVI reporter plasmid was a gift of Dr. Gabriel Núñez (University of Michigan Medical School).

### 2.5. Western Blot Analysis

Cells were washed with PBS and whole cell lysates were obtained by lysing them in RIPA lysis buffer (Santa Cruz Biotechnology, Santa Cruz, CA, USA). Protein concentration was determined by BCA following the manufacturer’s instructions (G Biosciences, St. Louis, MO, USA). Proteins (25 µg) were resolved by SDS-PAGE and transferred to PVDF filters (Cytiva, Little Chalfont Buckinghamshire, UK). Blots were incubated with antibodies against IkBɑ (Cell Signaling, Danvers, MA, USA), ß-catenin (Sigma, Burlington, MA, USA), NEDD8 (Y297) (AbCam, Cambridge, UK), and GSK-3, p65, GAPDH (0411) (Santa Cruz Biotechnology, Santa Cruz, CA, USA), and then incubated with an IgG kappa-binding protein or an anti-rabbit antibody conjugated with horseradish peroxidase (Santa Cruz Biotechnology, Santa Cruz, CA, USA). Bound protein was detected by a chemiluminescence assay (Cytiva, Little Chalfont Buckinghamshire, UK) in a LAS4000 mini imager (GE Healthcare, Little Chalfont Buckinghamshire, UK).

### 2.6. RT-qPCR

Total RNA was prepared using RNA-solv reagent (Omega Bio-Tek, Norcross, GA, USA). To assess mRNA expression, a reverse transcriptase–SYBR green quantitative polymerase chain reaction (RT-qPCR) method was employed. For the RT reaction, RNA (2 µg) was primed with random hexamers and reverse-transcribed with Superscript MMLV reverse transcriptase (Invitrogen, Carlsbad, CA, USA) in a 20 µL volume following the manufacturer’s instructions. The generated cDNA was subsequently analyzed for the expression levels of various genes through real time SYBR green PCR in an Applied Biosystems 7300 machine. The primers utilized for the analyzed genes are provided in the Supplementary Materials. The measurements were performed in triplicates and normalized against those of ß-actin as a housekeeping gene.

### 2.7. Transcriptome Analysis

PBMCs from five patients with chronic lymphocytic leukemia (CLL) were ex vivo treated with 250 nM MLN4924 (Cayman Chemical, Ann Arbor, MI, USA) and/or 1 µM CHIR-99021 (MedChemExpress, Sollentuna, Sweden) or an equivalent volume of dimethyl sulfoxide (DMSO) for 16 h. Total RNA was extracted using the E.Z.N.A HP Total RNA Kit (Omega Bio-Tek, Norcross, GA, USA) and subsequently sent to Eurofins Genomics (Ebersberg, Germany) for its analysis. Low-expressed genes were removed with CPM value less than 1. The abundance counts of each gene were then used to perform differential gene expression (DGE). DGE was performed using R/Bioconductor package edgeR package; edgeR normalizes by total count. The calcNormFactors function normalizes for RNA composition by finding a set of scaling factors for the library sizes that minimize the log-fold changes between the samples for most genes. Statistical tests were performed for each gene to compare the distributions between conditions (treatment vs. control), generating *p*-values for each gene. The final *p*-values were corrected by determining false discovery rates (FDR) using the Benjamin–Hochberg method, using an FDR corrected *p*-value (adjusted *p*-value).

### 2.8. RNA Immunoprecipitation Assay (RIP)

Briefly, PBMCs were isolated in a Ficoll gradient and fixed in 1% formaldehyde for 10 min. After washing with phosphate-buffered saline (PBS), the cells were resuspended in polysome lysis buffer (100 mM KCl, 5 mM MgCl_2_, 10 mM HEPES, pH 7.0, 0.5% Nonidet P40, 1 mM DTT, 100 U/mL RNAase inhibitor, and protease inhibitors (vanadate, aprotinin, and leupeptin)). The cells were centrifuged at full speed, and the supernatants were immunoprecipitated with an anti-eIF4A1 antibody (Santa Cruz Biotechnology, Santa Cruz, CA, USA) overnight at 4 °C. Pre-equilibrated protein G-agarose beads (ABT, Madrid, Spain) were then added and incubated for 4 h at 4 °C. After extensive washing, the crosslink was reversed by incubating in 0.3 M NaCl at 65 °C overnight and then digested with proteinase K for 3 h at 45 °C. RNA was then recovered using RNAsolv (Omega Bio-Tek, Norcross, GA, USA), precipitated, and resuspended in 10 µL of TE buffer. The entire recovered RNA was primed with random hexamers and reverse transcribed with Superscript MMLV reverse transcriptase (Invitrogen, Carlsbad, GA, USA) in a 20 µL volume following the manufacturer’s instructions. The bound RNAs were identified by real-time SYBR green PCR in an Applied Biosystems 7300 (Waltham, MA, USA) machine using the primers specified in the Supplementary Materials.

### 2.9. Luciferase Assays

HEK293T cells were cotransfected with 3 μg of the reporter constructs (TopFlash or pBVI) and 0.25 μg of pCMV-β-galactosidase in triplicate using the calcium phosphate technique. After 6 h of incubation, cells were washed and put back in fresh culture medium. 16 h later, the inhibitors were added to the cells. Luciferase and β-galactosidase activities were assessed 24–48 h later using luciferase substrate from Promega (Madison, WI, USA) and the Galacto-Light Plus system from Thermo Fisher Technologies (Bedford, MA, USA), respectively. The luciferase activities were normalized to β-galactosidase to obtain relative luciferase activity.

## 3. Results

### 3.1. Basal Expression of NF-kB Target Genes in CLL

**Figure 1:**
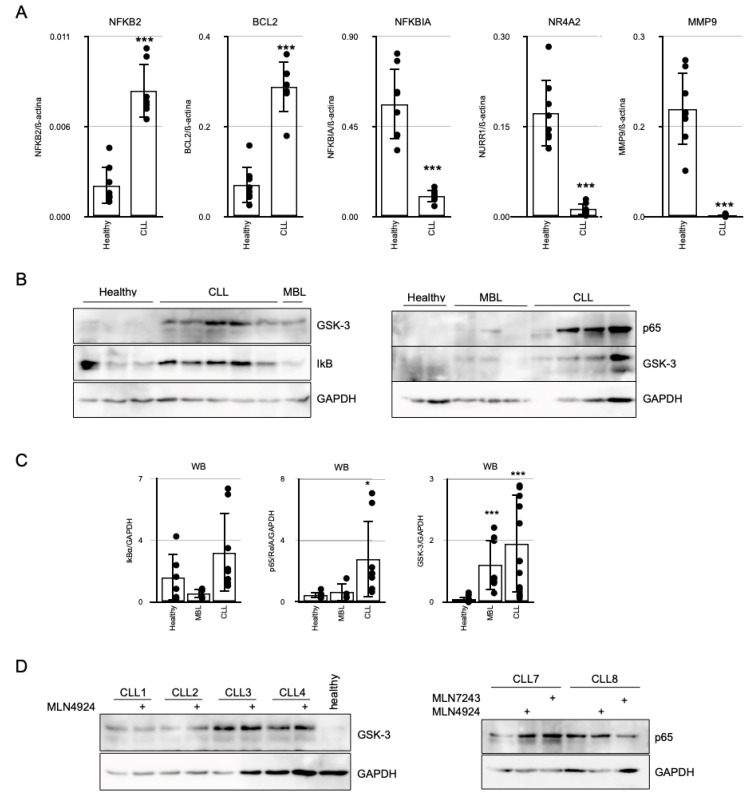
Basal expression of *NFKBIA* and its encoded protein, IkBα in CLL. (**A**) RT-qPCR analysis of the indicated genes in B cells from healthy and CLL donors (n = 7 each). Results are presented relative to ß-actin. Statistical significance was determined using Student’s *t*-test. *** *p* < 0.001, Student’s *t*-test. (**B**) Representative immunoblot analysis of whole cell extracts from chronic lymphocytic leukemia (CLL), monoclonal B-cell lymphocytosis (MBL) patients or healthy donors. (**C**) Densitometric analysis of the immunoblot bands for GSK-3, IkBα or p65/RelA relative to GAPDH in CLL or MBL patients or healthy donors. Groups were compared to healthy using Student’s *t*-test. * *p* < 0.05, *** *p* < 0.001, Student’s *t*-test. (**D**) Analysis of the effect of MLN4924 on the expression levels of GSK-3 and p65 proteins by western blot. PBMCs from CLL patients were treated ex vivo with 250 nM MLN4924 or 25 nM MLN7243/TAK243 for 24 h.

As previously noted, NF-kB is considered a pivotal signaling pathway in the survival of B-CLL cells. We analyzed the expression levels of several genes previously reported to be regulated by NF-kB in a small cohort of CLL patients. The messenger RNAs (mRNAs) of these genes accumulated at higher concentrations in peripheral blood mononuclear cells (PBMCs) from these patients compared to those obtained from healthy donors (Figure 1A). However, *MMP9*, *NR4A2* and notably *NFKBIA*, which encodes the inhibitor of the pathway IkBα but serves as a reliable indicator of NF-kB signaling, exhibited statistically significant lower expression in CLL samples (Figure 1A). Surprisingly, despite a relatively low mRNA expression level of NFKBIA, we observed an elevated accumulation of IkBα protein in CLL-PBMCs (Figure 1B,C). In addition to IkBα, we detected higher levels of p65 and GSK-3β proteins in samples from CLL patients compared to those obtained from healthy donors (Figure 1B,C). This observation was pertinent, as GSK-3β has been previously reported to selectively repress the expression of NF-kB target genes by phosphorylating p65 [5]. Furthermore, we also analyzed GSK-3 and p65 protein expression in samples from monoclonal B-cell lymphocytosis (MBL) patients. These patients exhibited elevated GSK-3 and p65 levels, albeit at lower levels compared to those observed in CLL patients (Figure 1B,C).

### 3.2. Translation Regulation and Ubiquitin-like Post-Translational Modifications Modulate NF-kB Signaling in CLL

Subsequently, we investigated the potential causes of the discrepancy between IkBɑ mRNA and protein levels in CLL. The constitutive NF-kB signaling in CLL could be associated with the overall increase in ubiquitin-like post-translational modifications observed in B-CLL cells [12,20]. Indeed, several studies support the NF-kB pathway as a primary target of MLN4924 in CLL cells, resulting in the stabilization of IkBα protein [9]. However, the role of NEDDylation in NF-kB signaling in B-CLL cells remains unclear, as they exhibit an elevated basal level of NEDDylation in conjunction with a high expression of IkBα. When B-CLL samples were treated with MLN4924 or the ubiquitin-activation enzyme inhibitor MLN7243/TAK243 ex vivo, we did not observe any variation in GSK-3 or p65 protein expression (Figure 1D). This response is markedly different from that observed in HEK293T cells, where MLN4924 induced a decline in transfected GSK-3ß or p65, while an increase in basal NEDDylation resulted in the accumulation of the endogenous proteins (Supplementary Figure S1). An RNA-seq analysis in B-CLL cells revealed a high number of NF-kB target genes that were significantly repressed by MLN4924 treatment (Supplementary Figure S2). However, some were induced, including *NURR1/NR4A2*. NURR1 has been associated with anti-inflammatory actions by impairing p65/RelA transactivation in microglia and astrocytes [6]. Such trans-repression necessitates p65 phosphorylation by GSK-3ß to tether NURR1. These findings suggest that, while NEDDylation significantly modulates IkBα protein accumulation, it does not affect the stability of p65 and GSK-3ß in CLL cells.

**Figure 2:**
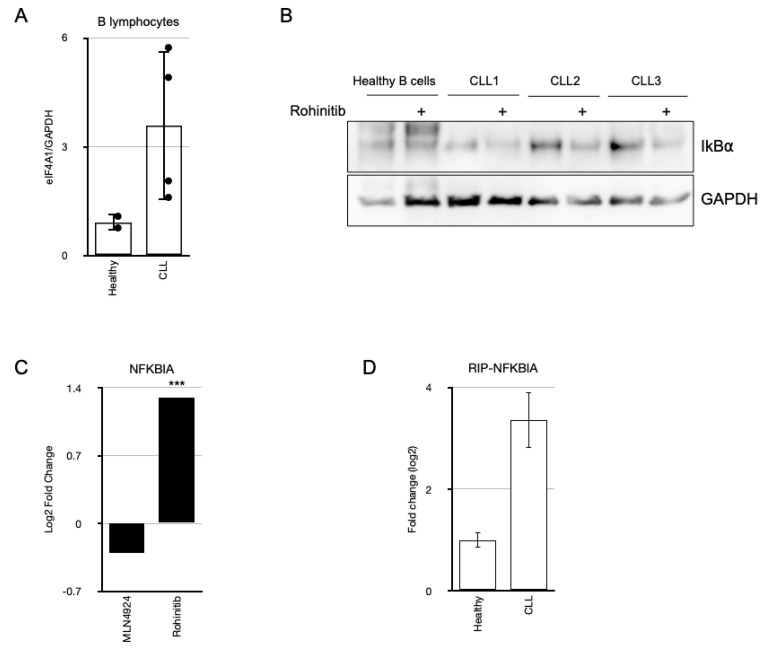
eIF4A1 overexpression in CLL cells and its regulation of IkBα expression. (**A**) eIF4A1 protein expression in B-CLL cells from CLL patients (n = 4) compared to that in B cells isolated from a healthy donor. Densitometric quantification of western blot bands is represented relative to the housekeeping gene GAPDH. (**B**) Western blot analysis of whole cell protein extracts obtained from PBMCs of three CLL patients and B lymphocytes isolated from a healthy donor treated ex vivo with 50 nM Rohinitib for 16 h. (**C**) NFKBIA mRNA expression levels in samples from five CLL patients treated for 16 h with 250 nM MLN4924 or 50 nM Rohinitib analyzed by NGS. (MLN4924 *p* = 0.334, rohinitib *p* = 0.000002). *** *p* < 0.001. (**D**) RNA immunoprecipitation assay showing the fold change binding variation of eIF4A1 to NFKBIA mRNA in CLL relative to healthy PBMCs. Measurements were done in triplicate.

Many tumors, including CLL, have been reported to be dependent on oncogene translation to survive and sustain their transformation [21]. To determine whether an alteration in *NFKBIA* translation was involved in the discrepancy between its messenger and protein levels, we treated B-CLL cells ex vivo with Rohinitib, an inhibitor of the eukaryotic translation initiation factor eIF4A1, which is overexpressed in CLL cells (Figure 2A) [17]. As shown in Figure 2B, Rohinitib decreased the accumulation of IkBα protein in B-CLL cells but not in B cells immuno-separated from a buffy coat of a healthy donor. In accordance with this, the inhibition of eIF4A1 induced the stabilization of the *NFKBIA* mRNA, blocking its translational-associated degradation, as previously reported to occur with other translationally-regulated genes in CLL [17] (Figure 2C). Furthermore, we could measure a higher binding of eIF4A1 to *NFKBIA* mRNA in CLL than in healthy-donor PBMCs, using an RNA immunoprecipitation assay (RIP) (Figure 2D). Consequently, the elevated translation of *NFKBIA* messenger in the clonal CLL cells would induce the accumulation of IkBα and the disruption of the negative feedback of the pathway.

### 3.3. B-CLL Cells Do Not Activate NF-kB Signaling in Response to GSK-3ß Inhibition

**Figure 3:**
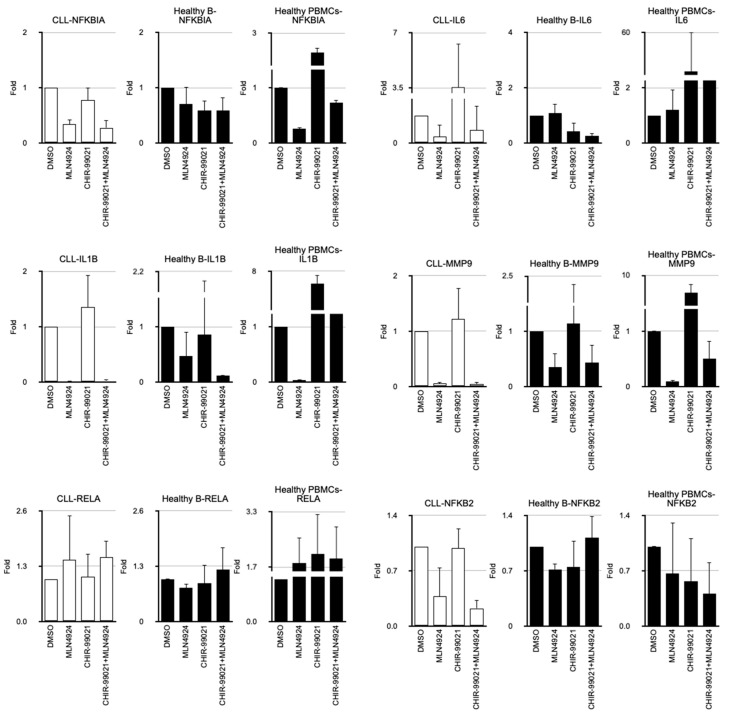
B-CLL cells do not activate NF-kB target genes in response to CHIR-99021. RT-qPCR analysis of the indicated mRNAs in peripheral blood mononuclear cells (PBMCs) from CLL patients (n = 3) or pools of healthy donors (n = 2) as well as in isolated healthy B cells from buffy coats (n = 3), treated ex vivo with 250 nM MLN4924, 1 µM CHIR-99021 or a combination of both for 24 h. The mean of the fold variations and the corresponding standard deviations are represented. Some histograms were cropped to maintain the scale for comparison purposes.

GSK-3ß activity has been reported to restrain NF-kB signaling in different tumors, but its role in CLL remains unclear [22]. We demonstrated that CHIR-99021 induced NF-κB activity in HEK293T cells, while MLN4924 repressed it (Supplementary Figure S1). Given that GSK-3β and p65 are highly expressed in CLL cells, we investigated whether this regulation is also active in them and how NEDDylation may modulate it. We focused on the expression of a group of well-known NF-κB target genes. When PBMCs from healthy donors were treated with the GSK-3ß inhibitor CHIR-99021, the expression of genes like *IL6*, *IL1B*, *MMP9*, and *NFKBIA* was strongly induced (Figure 3, Healthy PBMCs panels). Conversely, MLN4924 blocked both the basal and CHIR-99021-induced expression of these genes (Figure 3, Healthy PBMC panels). The expression of other genes involved in NF-κB signaling, such as *RELA* or *NFKB2*, was largely unaffected by either treatment. Since B cells constitute only a small fraction of healthy PBMCs, we isolated B cells from buffy coats of healthy donors. Surprisingly, CHIR-99021 did not induce these NF-kB target genes in healthy B cells but rather repressed them in some cases, while MLN4924 had little or no effect (Figure 3, Healthy B panels). When we analyzed the response to these treatments in PBMCs from CLL patients, comprising over 80% by clonal B cells, we found that CHIR-99021 had essentially no effect on these genes (Figure 3, CLL panels). However, B-CLL cells acquired the sensitivity to MLN4924 of their basal expression observed in PBMCs from healthy donors. These findings discard the activation of the survival NF-κB pathway by GSK-3β inhibition in CLL cells.

### 3.4. MLN4924 and CHIR-99021 Synergistically Enhance Their Individual Effects on B-CLL Cell Viability

**Figure 4:**
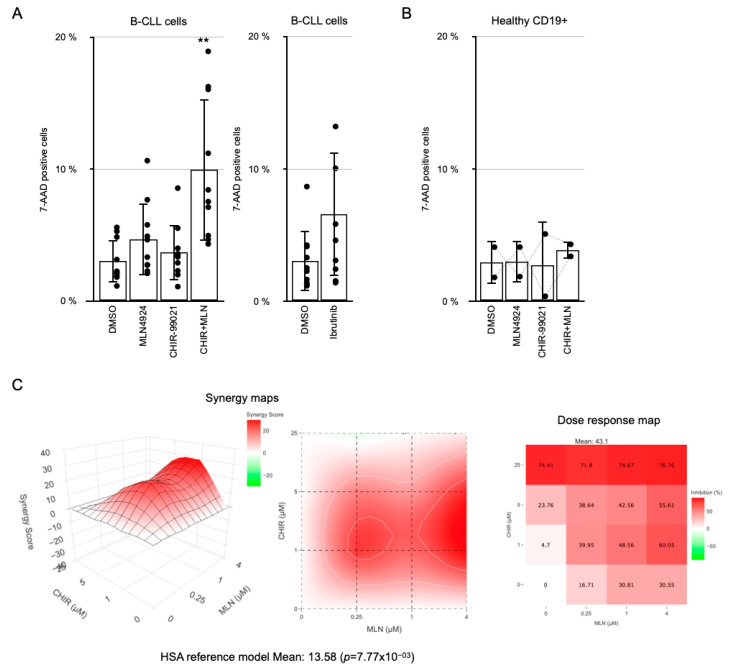
Combinational induction of B-CLL cell death by CHIR-99021 and MLN4924. (**A**) Patient B-CLL (n = 11) cell death analysis after the combinational treatment with the GSK-3ß inhibitor CHIR-99021 (1 µM), and the NAE inhibitor MLN4924 (250 nM) measured after 24 h as 7-AAD stained cells by flow cytometry. Response of B-CLL cells from patients (n = 10) to 10 µM Ibrutinib, a standard-of-care therapy, under the same conditions is shown for comparison. Groups were compared to DMSO-treated using Student’s *t*-test. ** *p* < 0.01, Student’s *t*-test. (**B**) Cell death induction of the combined treatment with 1 µM CHIR-99021 and 250 nM MLN4924 for 24 h over CD19-positive peripheral blood cells obtained from two different pools of healthy donors, measured as 7-AAD-stained cells. (**C**) Cell viability of B-CLL cells in response to increasing doses of CHIR-99021 and MLN4924 was determined by an XTT assay. The results were analyzed for the synergy score at the SynergyFinder+ web page (https://synergyfinder.org). Representative results of 3 different assays are shown.

It has been previously reported that GSK-3ß inhibition induces apoptosis in CLL cells [7]. Given that we observed that B-CLL cells express high levels of GSK-3, and our data strongly suggest that it does not restrain their NF-κB activity, we sought to explore the combinatorial effect of both GSK-3β and NEDDylation inhibition in these cells. CHIR-99021/Laduviglusib induced a modest increase in B-CLL cell death on its own. However, when combined with MLN4924, cell death was induced threefold after 24 h, and it was higher than the sum of the effects of the two inhibitors individually (Figure 4A). The average cell death reached was higher than that induced by the standard-of-care drug Ibrutinib under the same conditions (Figure 4A). Notably, when we tested this combination on two pools of healthy donor CD19+ cells, no increase in cell death was observed (Figure 4B). A study of synergy between these two drugs in B-CLL cells from different patients showed a significant score, although with notable differences among patients (Figure 4C). We could also observe some degree of synergy between CHIR-99021 and the NF-κB-signaling inhibitor BAY11-7082 (Supplementary Figure S3) [23].

### 3.5. MLN4924 Potentiates the Inhibition of BCL-2 mRNA Expression Induced by CHIR-99021

**Figure 5:**
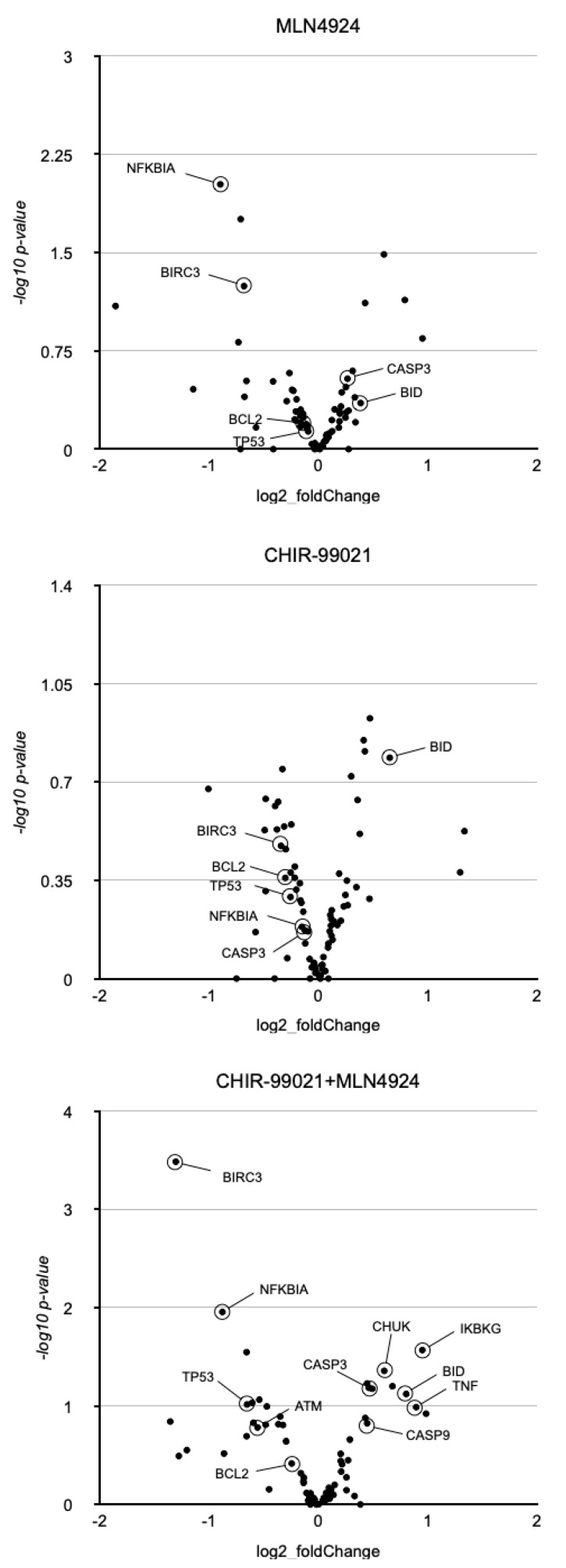
Apoptosis-related gene expression variations in response to MLN4924 and CHIR-99021 in B-CLL cells. A transcriptome analysis was conducted in PBMCs from five CLL patients to identify genes that could be mediating the enhanced cell death induced by the combination of CHIR-99021 (1 µM) and MLN4924 (250 nM). Volcano plots of the mean variations in apoptosis-related genes after 24 h, compared to vehicle treated cells (log2 fold change) obtained with each ex vivo treatment are depicted.

**Figure 6:**
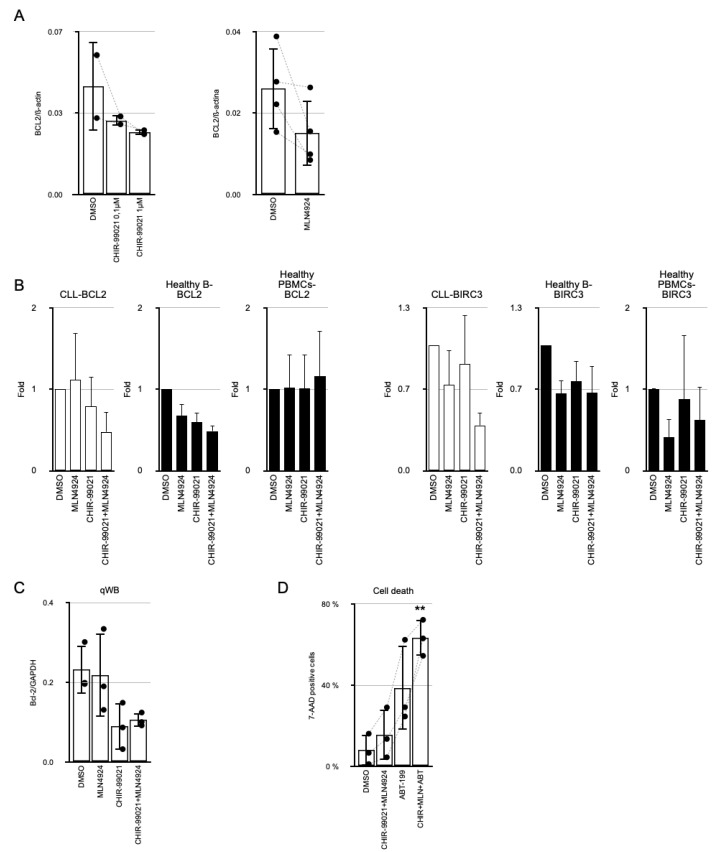
MLN4924 collaborates with CHIR-99021 in the repression of *BCL2* and *BIRC3*. (**A**) Analysis of the inhibitory effect of either CHIR-99021, at 0.1 and 1 µM (n = 2), or MLN4924 at 250 nM (n = 4) over *BCL2* mRNA expression in B-CLL cells by RT-qPCR. (**B**) RT-qPCR analysis of BCL2 and *BIRC3* messenger RNA expression in B cells from CLL patients (n = 7 and n = 3, respectively) treated ex vivo with 250 nM MLN4924, 1 µM CHIR-99021, or a combination of both for 24 h, relativized to ß-actin mRNA expression. Expression levels in isolated B lymphocytes from three distinct healthy donors or PBMCs from three pools of healthy donors are presented at the same scale for comparative purposes. (**C**) Densitometric analysis of three western blots demonstrating the impact of 250 nM MLN4924 and/or 1 µM CHIR-99021 on the accumulation of BCL-2 protein in B-CLL cells from three CLL patients. (**D**) Analysis of the induction of cell death by ABT-199/Venetoclax at 1 nm in combination or not with 1 µM CHIR-99021 and 250 nM MLN4924. Cell death was assessed by 7-AAD staining and subsequent flow cytometry analysis. Groups were compared to DMSO-treated cells using Student’s *t*-test in all experiments. ** *p* < 0.01, Student’s *t*-test.

To elucidate the molecular mechanisms underlying the potentiation effect, we conducted a comprehensive analysis of mRNA expression variations following the treatments. We performed a whole transcriptome analysis on samples obtained from five patients who underwent ex vivo treatment. Supplementary Figure S4 provides a general overview of how the various treatments influence the expression of WNT- and NF-kB-target genes. Given the association between GSK-3 inhibition and an increase in apoptosis in CLL, we focused on apoptosis-related genes that were cooperatively affected by CHIR-99021 and MLN4924. The screening revealed significant effects on relevant genes such as *BCL2*, *TP53*, *BIRC3*, *ATM*, *FAS*, *FASLG*, *Caspases 8*, *9* and *3*, or *BID* (Figure 5). *BCL2* gene repression by GSK-3β inhibitors has previously been reported in CLL, and we could confirm this observation [7]. Additionally, we observed a reduction in *BCL2* mRNA accumulation in response to MLN4924 (Figure 6A). This gene plays a pivotal role in the survival and resistance of B-CLL cells, being the most expressed anti-apoptotic gene (Supplementary Figure S5). Surprisingly, *BIRC3* was also among the most expressed genes in B-CLL cells. The combined treatment of MLN4924 and CHIR-99021 reduced the mRNA and protein expression of both *BCL2* and *BIRC3* by half (Figure 5 and Figure 6B). Although the response of *BCL2* in CLL and healthy B cells was similar, the reduction in *BIRC3* expression was not observed or was significantly milder in the latter. Since the combined actions of CHIR-99021 and MLN4924 reduced the mRNA and protein expression of *BCL2* in B-CLL cells (Figure 6B,C), we investigated whether this could enhance the efficacy of inhibitors of BCL-2 activity. We utilized sub-optimal doses of the potent BCL-2 inhibitor ABT-199/Venetoclax. At a 1 nM dosage of ABT-199, pretreatment with the combination of CHIR-99021 and MLN4924 significantly potentiated its cytotoxic effect (Figure 6D).

## 4. Discussion

NF-kB is a pivotal pathway in cancer cell biology, regulating the transcriptional activation of genes involved in processes such as inflammation and survival. The constitutive activation of the NF-kB pathway has been associated with the prolonged lifespan of B-CLL tumor cells. However, several observations challenge the notion of constitutive activation of NF-kB signaling in CLL cells. Utilizing PBMCs obtained from healthy donors as a reference, we confirmed the elevated expression of genes previously associated with constitutive activation of NF-kB in CLL cells, including *BCL2* [7].^.^ Although PBMCs from healthy donors exhibit a lower percentage of B cells compared to those from CLL patients, they serve as a comparative measure to highlight the relative differences in the expression of the analyzed genes. Notably, the expression of other NF-kB target genes, such as *NFKBIA*, which encodes for IkBα, remained relatively low. Furthermore, employing healthy PBMCs as a control, we observed higher levels of IkBα protein expression in CLL PBMCs, suggesting a discrepancy between the mRNA and protein-expression levels of IkBα in B-CLL cells. The stability of IkB proteins is pivotal in the activation of the canonical NF-kB pathway. Inactivating mutations in members of this family of inhibitors have been associated with a poorer prognosis in CLL [24]. Since the degradation of IkBα is mediated by Cullin RING ubiquitin ligases (CRL), MLN4924/Pevonedistat has demonstrated the capacity to stabilize it, thereby impairing NF-kB signaling in CLL and in diffuse large B-cell lymphoma [9,10]. Nevertheless, given that B-CLL cells exhibit higher ubiquitin-like post-translational modification levels compared to healthy CD19+ B cells [12], their elevated IkBα accumulation is surprising, suggesting alternative regulatory mechanisms. To address this, we explored a potential translational control of the *NFKBIA* messenger. An overall increment in mRNA translation has been reported in CLL and other tumors, associated with the overexpression of the translation initiation factor eIF4A1 [25]. This phenomenon has been reported in CLL for p27 and MYC [26], and it is an active area of therapeutic investigation in this pathology, as it affects key proliferation-related proteins [27]. We observed that RNA translation inhibition with Rohinitib resulted in a decrease in the levels of IkBɑ protein and a protection from the translation-associated degradation of its mRNA, as previously reported for *MYC* mRNA [17]. Furthermore, an RNA immunoprecipitation assay demonstrated a higher amount of eIF4A1 bound to *NFKBIA* mRNA in CLL B lymphocytes. *NFKBIA* translational regulation has been previously reported in mouse embryonic fibroblasts through the phosphorylation of another initiation factor: eIF4E [14]. However, to the best of our knowledge, this is the first time that IkBα accumulation is associated with eIF4A1 overexpression in B-CLL, providing an explanation for the discrepancy between its mRNA and protein levels. The reasons behind the selection of this anti-survival mechanism in a tumor and its evolution as the malignancy progresses remain intriguing subjects for further research.

In contrast, we observed higher expression of the proteins GSK-3ß and p65 in B-CLL cells. We investigated whether the high intrinsic NEDDylation of CLL cells was associated with their elevated levels of GSK-3ß and p65 proteins [12,13]. Although MLN4924 induced a decline in exogenously expressed GSK-3ß or p65 in HEK293T cells, we were unable to replicate this effect in primary B-CLL cells treated ex vivo. GSK-3ß has been reported to turn p65 into a transcriptional repressor by its phosphorylation at serine 468, among others [5]. We could demonstrate this mechanism in HEK293 cells. The induction of NF-kB target genes by GSK-3ß inhibition with CHIR-99021 suggested that it was also active in healthy PBMCs. In mantle cell lymphoma and in diffuse large B-cell lymphoma, WNT/ß-catenin signaling participates in NF-kB-mediated transcription [28,29]. However, although CHIR-99021 induces ß-catenin accumulation (as shown in the Supplementary Figure S6), its induction of pro-survival NF-kB target genes appears defective in B-CLL cells. The elevated translation of the *NFKBIA* mRNA we observed in B-CLL cells, acting downstream of GSK-3ß and p65 transcriptional repression, may explain their lack of NF-kB signaling induction by CHIR-99021.

Subsequently, we investigated whether the combinatorial inhibition of GSK-3β and NEDDylation could synergistically induce B-CLL cell death. The induction of B-CLL cell death by inhibition of GSK-3 activity has been a subject of controversy, as it was also reported by Ougolkov et al. [7], while Lu et al. showed an enhancement of B-CLL cell survival after SB-216763 treatment [30]. Our experiments revealed a stronger cell death induction in ex vivo treatment of PBMCs from CLL patients with the combination of CHIR-99021/Laduviglusib and MLN4924/Pevonedistat compared to each compound used separately. The distinct bypass of the transrepression exerted by the tandem p65-GSK-3ß through a higher basal translation in B-CLL cells and the subsequent stabilization of the IkBα protein induced by ubiquitination inhibition appear to contribute to this collaboration, as evidenced by our findings. However, it is plausible that other factors may also be involved.

Overexpression of *BCL2* is a hallmark of B-CLL cells (Supplementary Figure S6), which contributes to their extended lifespan and serves as a primary target of current therapeutic interventions. Previous studies have demonstrated that GSK-3 inhibition in CLL cells results in a reduction in *BCL2* mRNA expression through an epigenetic mechanism, potentially mediating its induction of apoptosis [7]. In this study, we demonstrate how MLN4924 enhances such *BCL2* inhibition. Notably, we observed this repression also in isolated healthy B cells, but not in the entire PBMC fraction (primarily composed of T cells), suggesting specificity for B lymphocytes. The absence of induction by CHIR-99021 and the mild repression by MLN4924, which is consistent with bona fide NF-kB target genes, imply that *BCL2* is not a target of NF-KB in B-CLL lymphocytes. Most interestingly, CHIR-99021 and MLN4924 potentiate the action of ABT-199/Venetoclax. Given that these agents inhibit BCL-2 action through distinct mechanisms, it is intriguing to consider their potential application in cases of resistance to Venetoclax or to reduce its doses to minimize adverse effects. Our NGS transcriptome analysis unveiled additional potential mediators of the cytotoxic effect of the combinational treatment with MLN4924 and CHIR-99021. Several caspase genes (3, 7, 8 and 9) and the proapoptotic genes *BIK* and FASLG exhibited an accumulation of their corresponding messenger RNAs. Conversely, mRNAs of genes involved in apoptosis induction, such as *BIRC3*, *TP53* and *ATM*, were downregulated. Others, like *TRAF2*, *CHUK*, or *TNF* experimented a strong induction after the combined GSK-3ß and NAE1 inhibition. The case of *BIRC3* is particularly noteworthy, as it has been reported to inhibit apoptosis in other tumors [31]. However, low levels or inactivating mutations of BIRC3 have been associated with unfavorable prognosis in CLL [32]. Inactivating mutations of *BIRC3* have been identified in 4% of CLL patients. In CLL, BIRC3 has been implicated in the degradation of NIK, thereby reducing NF-kB non-canonical signaling. Notably, the combinational treatment with CHIR-99021 and MLN4924 induced the repression of *BIRC3*. Low *BIRC3* levels have been associated with elevated *BCL2* expression through the activation of NF-kB signaling and heightened sensitivity to Venetoclax [33]. However, our experiments did not demonstrate such crosstalk between *BIRC3* and *BCL2.* Although the reduction in *BIRC3* mRNA expression aligns with increased sensitivity to Venetoclax, this does not appear to be attributable to an increase in *BCL2* mRNA or protein levels.

## 5. Conclusions

In summary, we present herein a previously uncharacterized mRNA translation regulation mechanism of IkB in B-CLL cells that elucidates the absence of correlation between its mRNA and protein levels. This finding provides rationale for the lack of NF-kB signaling induction by GSK-3ß inhibitors in these cells. We observed synergy in the combinational treatment of B-CLL cells ex vivo with CHIR-99021 and MLN4924. Furthermore, we provide evidence of collaborative BCL2 expression inhibition by this combined treatment, offering some mechanistic support for its cytotoxic action.

## Figures and Tables

**Table 1:** Characteristics of patients.

	Age (Years)	Gender	IgHV	CTG	NGS Mutation
CLL-1	65	Male	Unmutated	Del13q14	XPO1
CLL-2	61	Female	Mutated	Del13q14	FBXW7
CLL-3	91	Male	ND	ND	ND
CLL-4	77	Male	ND	ND	ND
CLL-5	72	Female	Mutated	Del13q14	No alterations
CLL-6	55	Male	Unmutated	Normal	No alterations
CLL-7	86	Male	Mutated	Normal	No alterations
CLL-8	80	Female	ND	ND	ND
CLL-9	71	Female	Unmutated	Normal	NOTCH1, SF3B1
CLL-10	81	Male	Mutated	Normal	ND
CLL-11	92	Female	Unmutated	ND	ND
CLL-12	78	Male	ND	Del11q23 and Del13q14	ND
CLL-13	61	Male	ND	Del13q14	ND
CLL-14	73	Female	ND	ND	ND
CLL-15	80	Female	ND	ND	ND
CLL-16	54	Male	Unmutated	Normal	TP53, NOTCH1, BIRC3 y XPO1

ND: Not determined.

## Data Availability

All data are available from the corresponding authors upon reasonable request.

## Supplementary Materials

The following supporting information can be downloaded at: https://www.mdpi.com/article/10.3390/cancers17030533/s1, Figure S1: NEDDylation regulates GSK3ß and p65 stability in HEK293T cells; Figure S2: Sensitivity of NF-kB target genes to MLN4924 in CLL; Figure S3: Synergy score of CHIR-99021 and BAY11-7082 treatment over B-CLL cells ex vivo; Figure S4: Transcriptome analysis of the transcriptional changes induced by MLN4924 and CHIR-99021 in CLL; Figure S5: Gene expression in CLL; Figure S6: CHIR-99021 induces the accumulation of ß-catenin in B-CLL cell.; Table S1: RT-qPCR primers.

## Abbreviations

CLL: Chronic lymphocytic leukemia; NEDD8: Neural precursor cell expressed, developmentally down-regulated 8; B-CLL: chronic lymphocytic leukemia B cell; GSK-3ß: Glycogen synthase kinase-3 beta; PBMCs: Peripheral blood mononuclear cells; RIP: RNA immunoprecipitation assay.
