# Supplementary material for: Distinct NF-kB Regulation Favors a Synergic Action of Pevonedistat and Laduviglusib in B-Chronic Lymphocytic Leukemia Cells Ex Vivo
Source: Cancers (Basel). 2025 Feb 5;17(3):533. doi: 10.3390/cancers17030533 (PMC11816723; doi:10.3390/cancers17030533)
Supplement: Supplementary file 1 [file cancers-17-00533-s001.zip › cancers-3411940-supplementary.pdf]

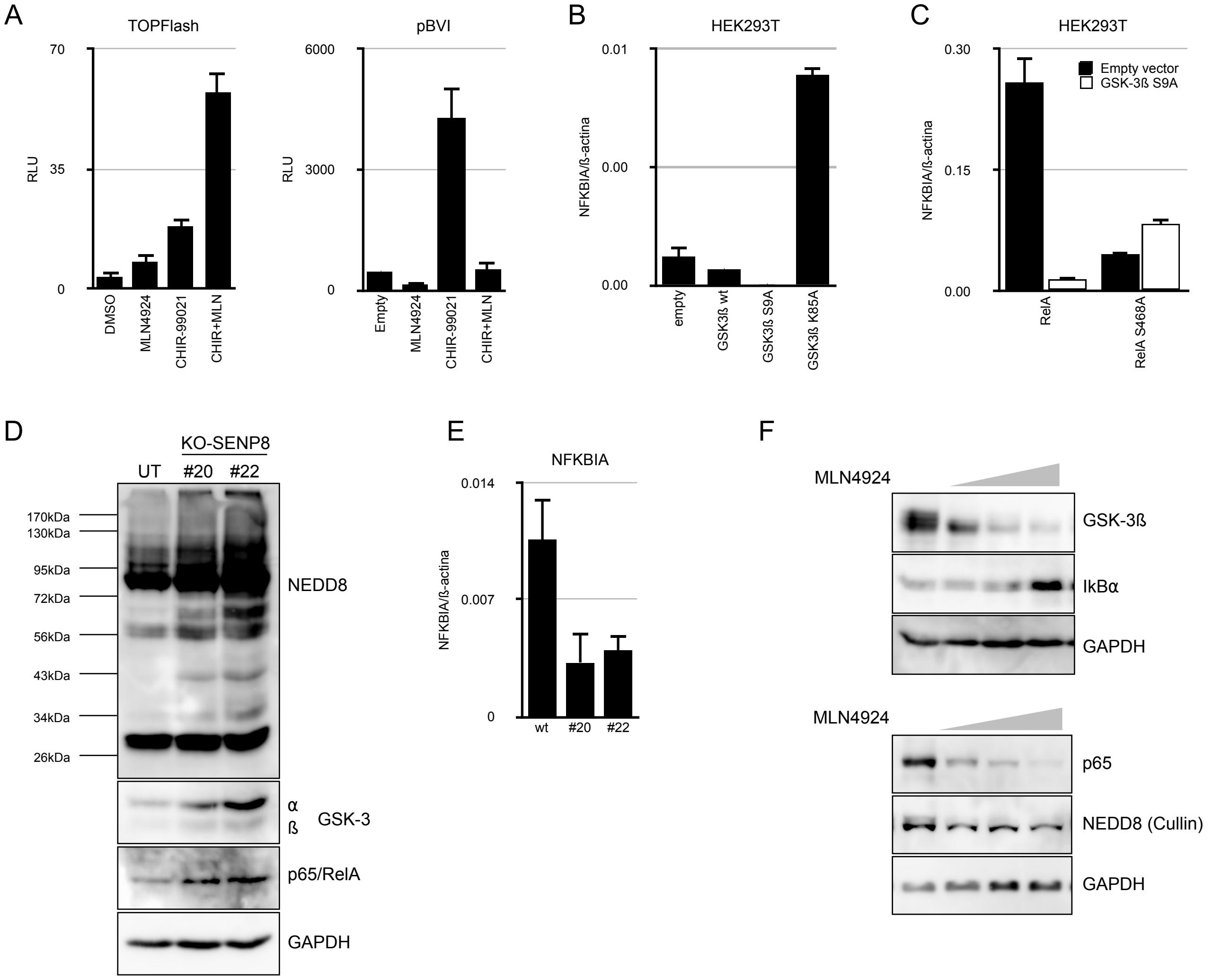

Figure S1. NEDDylation regulates GSK3ß and p65 stability in HEK293T cells.

A. Luciferase assays showing the impact of MLN4924 or CHIR-99021 on WNT or NF-kB signaling, assessed using the TopFlash or pBVI reporter plasmids, respectively. Cells were treated with 250 nM of MLN4924 and/or 1 µM of CHIR-99021 for 24 hours. Each measurement was done in triplicate and normalized to transfected ß-galactosidase activity (relative luciferase units, RLU).

B. Transfection of a dominant negative mutant of GSK-3ß (K85A) induces accumulation of endogenous *NFKBIA* mRNA in HEK293T cells, while wt GSK-3ß or the serine to alanine 9 mutant (S9A) represses it, as analyzed by RT-qPCR and normalized to ß-actin expression.

C. Transfection of a serine to alanine 468 mutant of RelA/p65 (S468A) renders HEK293T cells refractory to the inhibition of endogenous *NFKBIA* mRNA expression by GSK-3ß S9A. mRNA expression analysis was conducted by RT-qPCR as in B.

D. SENP8 gene were deleted from HEK293T cells by transfecting two different commercial CRISPR/Cas9 constructs for SENP8/DENP1 (pCMV Cas9-GFP SENP8 #20 and #22) and sorting of the green fluorescent cells. An increase in general NEDDylation was observed by western blot using an antibody against NEDD8, when compared to untransfected cells (UT). Levels of GSK-3 and p65/RelA were analyzed in the same blot.

E. KO of SENP8 in HEK293T cells is accompanied by a reduction in *NFKBIA* mRNA expression, as analyzed by RT-qPCR. Data show measurements in triplicate and relative to-actin levels.

F. HEK293T cells were treated with 62.5, 125 and 250 nM MLN4924 for 24 hours and the indicated proteins were analyzed by western blotting. Cullin NEDDylation is shown as a control.

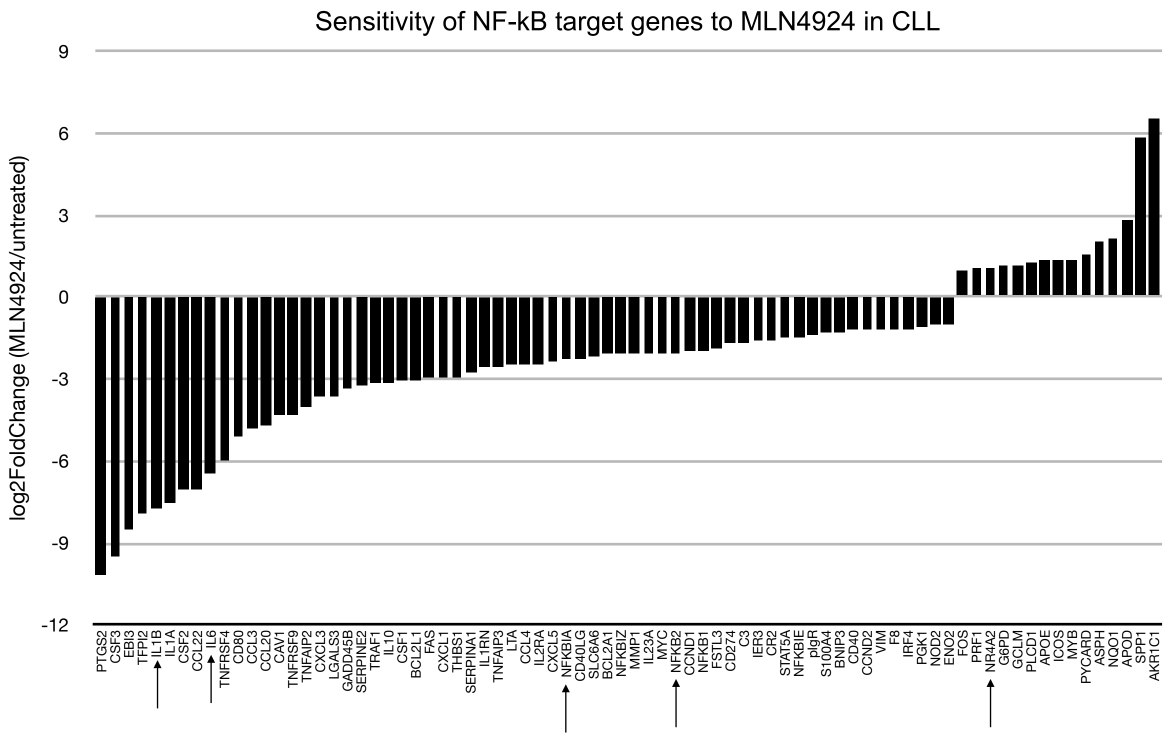

Figure S2. Sensitivity of NF-kB target genes to MLN4924 in CLL.
Distribution of the sensitivity to 0.25µM MLN4924 of NF-kB target genes in B-CLL cells.

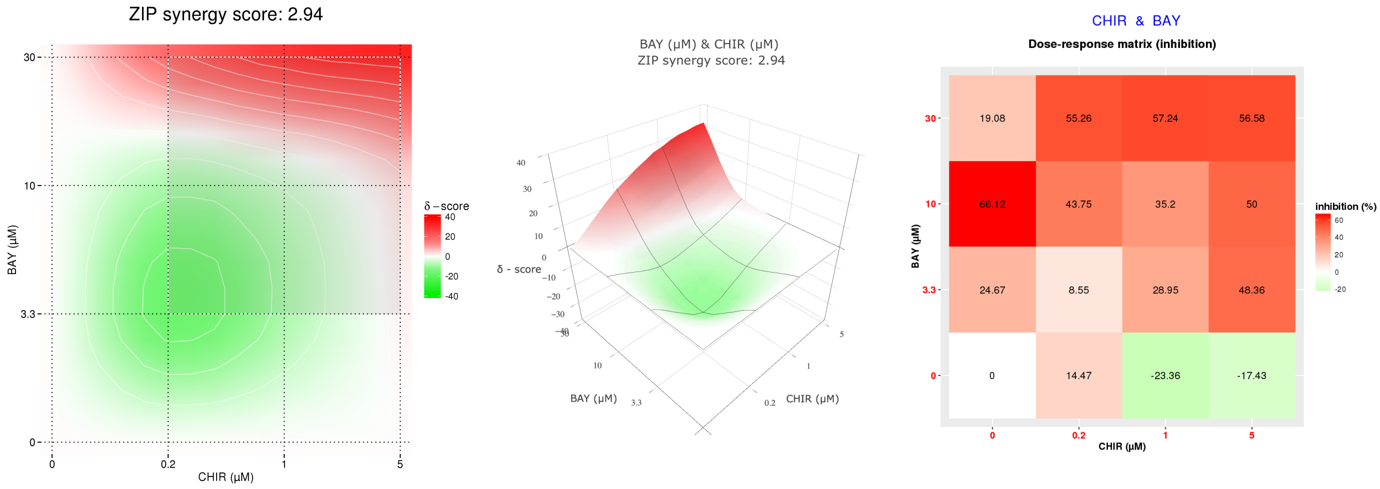

Figure S3. Synergy score of CHIR-99021 and BAY11-7082 treatment over B-CLL cells ex vivo.
Cell viability in response to increasing doses of CHIR-99021 and BAY11-7082 was determined by an XTT assay. The synergistic effect of both drugs was analyzed using the SynergyFinder+ web platform. Representative results of three different assays are presented.

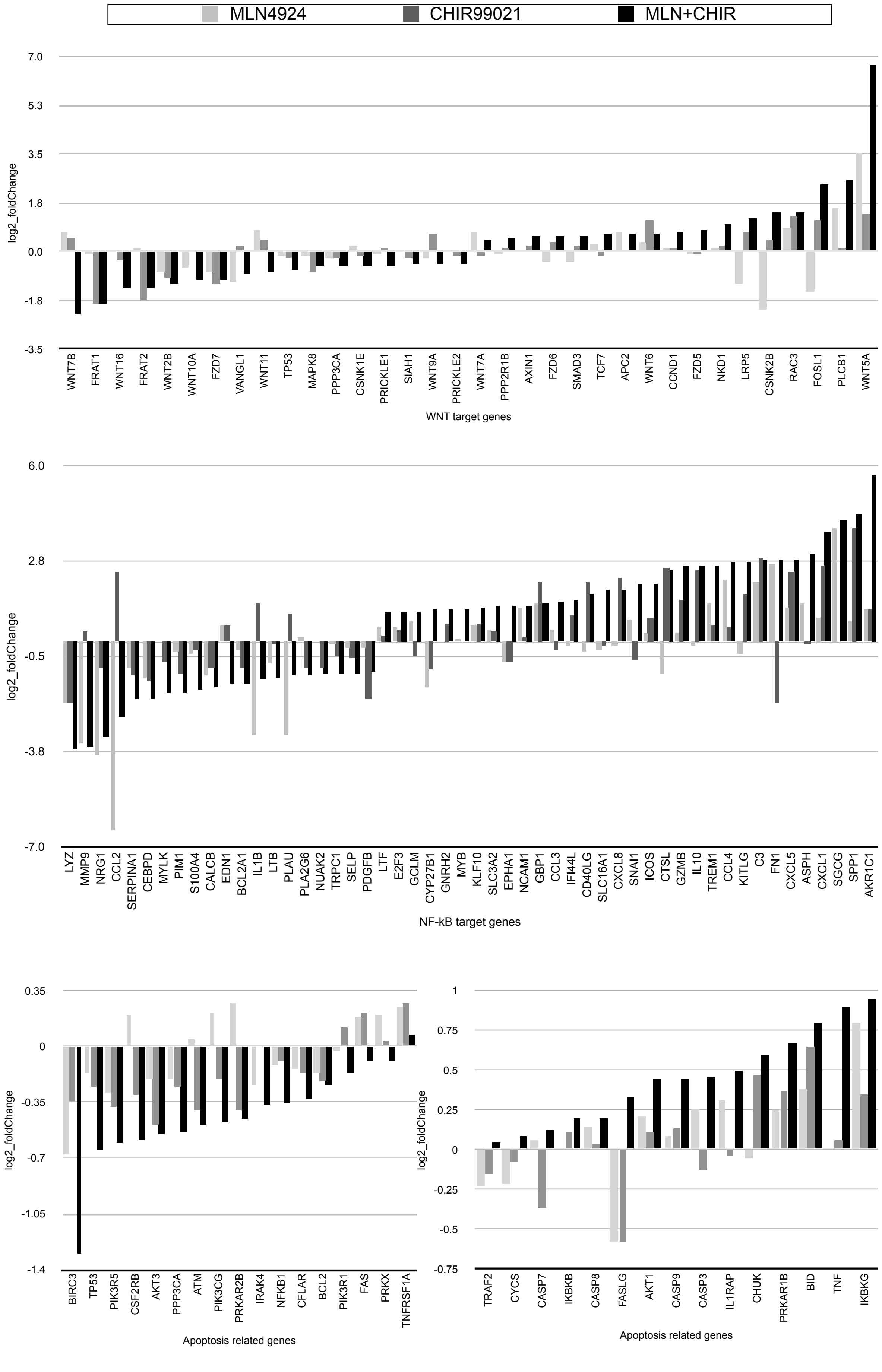

Figure S4. Transcriptome analysis of the transcriptional changes induced by MLN4924 and CHIR-99021 in CLL. Average variations in mRNA expression of WNT, NF-kB and apoptosis signaling target genes in B-CLL cells from five CLL patients induced by 250nM MLN4924, 1µM CHIR-99021 or both for 24 hours, relative to untreated cells.

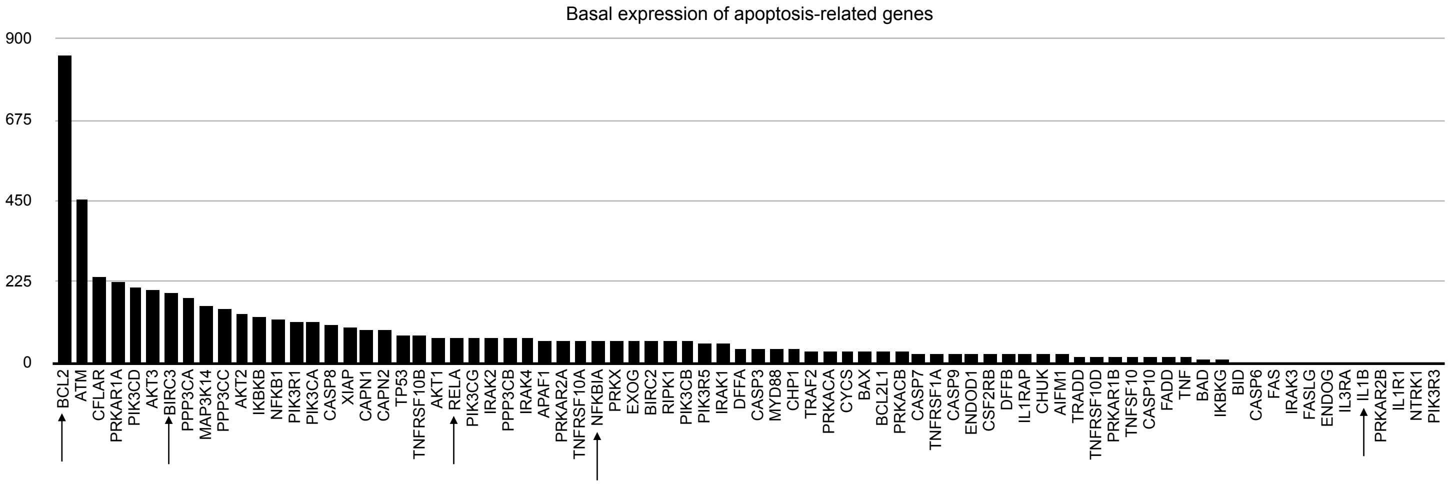

Figure S5. Gene expression in CLL.
Average basal expression of apoptosis-related genes in B cells from the five CLL patients studied by RNA-sequencing.

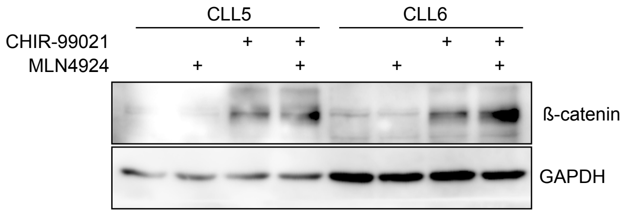

Figure S6. CHIR-99021 induces the accumulation of ß-catenin in B-CLL cells.
Immunoblot analysis of ß-catenin expression in B-CLL cells treated ex-vivo with 250 nM MLN4924 and/or 1µM CHIR-99021 for 16 hours.

RT-qPCR primers

Gene	Forward and reverse primers (5′-3′ sequence)
NFKBIA	AAGTGATCCGCCAGGTGAAG<br>CTGCTCACAGGCAAGGTGTA
IL6	TCTCCACAAGCGCCTTCG<br>GCCTCTTTGCTGCTTTCACA
IL1B	TCATTGCTCAAGTGTCTGAAGC<br>TGGTCGGAGATTCGTAGC
NFKB2	TGGCCGGGACAAGAGAAAAG<br>CGGAAGCCTCTCTGCTTAGG
NR4A2 (NURR1)	CTGTAACTCGGCTGAAGCCAT<br>AGGGGCATTTGGTACAAGCA
MMP9	TTCAGGGAGACGCCCATTTC<br>TGGGTGTAGAGTCTCTCGCT
BCL2	GGATAACGGAGGCTGGGATGCC<br>TTGGGGCAGGCATGTTGACTTCAC
BIRC3	TCGCTTGAAAAGACTGGGCT<br>ATGTGCCAGTAGGAGACTGC
ß-actin	GCGGGAAATCGTGCGTGACATT<br>GATGGAGTTGAAGGTAGTTTCGTG
